# “*Help Us!*”: a content analysis of COVID-19 help-seeking posts on Weibo during the first lockdown

**DOI:** 10.1186/s12889-023-15578-y

**Published:** 2023-04-19

**Authors:** Yu Guo, Yongkang Hou, Hongzhe Xiang, Liang Chen

**Affiliations:** 1 Faculty of Humanities and Arts, Macau University of Science and Technology, R309, Wailong Avenida, Macau SAR Taipa, China; 2 School of Journalism and Communication, Tsinghua University, Beijing, China

**Keywords:** COVID-19, Online help-seeking, Public engagement, Lockdown, Social media

## Abstract

**Background:**

Social media is playing an increasingly important role in public emergencies for help-seekers, especially during the global COVID-19 pandemic. Wuhan, China, firstly official reported COVID-19 cases and implemented lockdown measures to prevent the spread of the virus. People during the first lockdown were restricted from seeking help face-to-face. Social media is more prominent as an online tool for people seeking help, especially for patients, than in other stages of the COVID-19 pandemic.

**Objective:**

This study aimed to explore the urgent needs presented in help-seeking posts in Wuhan during the first COVID-19 lockdown, the content features of these posts, and how they influenced online user engagement.

**Methods:**

This study collected posts from Weibo posted with specific help tags during the first COVID-19 lockdown in Wuhan: from 23 January 2020 to 24 March 2020, and eventually received 2055 data, including textual content, comments, retweets, and publishing location. Content analysis was conducted, and manual coding was performed on help-seeking typology, narrative mode, narrative subject, and emotional valence.

**Results:**

The result showed that help-seeking posts primarily were seeking medical (97.7%). Features of these posts were mainly adopting a mixed narrative mode (46.4%), released by relatives of patients (61.7%), and expressing negative emotions (93.2%). Chi-square tests suggested that help-seeking posts with mixed narrative modes released by relatives express more frequent negative emotions. Results of negative binomial regression indicated posts of seeking information (B = 0.52, *p* < .001,* e*^0.52^ = 1.68), with mixed narrative mode (B = 0.63, *p* < .001, *e*^0.63^ = 1.86), released by themselves (as referential groups) and with neutral emotions increased comments. Posts of seeking medical (B = 0.57, *p* < .01,* e*^0.57^ = 1.77), with mixed narrative mode (B = 1.88, *p* < .001, *e*^1.88^ = 6.53), released by people of unrelated patients (B = 0.47, *p* < .001, *e*^0.47^ = 1.60) and with neutral emotions increased retweets.

**Conclusions:**

This study provides evidence of what actual public demands are to be considered and addressed by governments and public administrators before implementing closure and lockdown policies to limit the spread of the virus. Meanwhile, our findings offer strategies for people help-seeking on social media in similar public health emergencies.

**Supplementary Information:**

The online version contains supplementary material available at 10.1186/s12889-023-15578-y.

## Background

As of March 2023, the global number of confirmed cases of COVID-19 surpassed 759 million, including more than 6.8 million deaths [1]. The high number of cases and fatalities serve as a sobering reminder that the fight against this public health crisis is far from over. Over the past three years, many countries have employed various measures to prevent the spread of the virus [2]. Different from many Western countries' voluntary control measures, the Chinese government tends to adopt the city lockdown strategy. This strategy is a highly centralized and collective approach to coping with human-to-human infectious diseases but involves restricting personal liberties. Undeniably, it is a controversial method of intervention. Wuhan was the first city to officially report the group of known COVID-19 cases [3], and it was also the first city to adopt a strict lockdown to cope with this unknown virus, which was started on 23 January 2020, to 8 April 2020. The lockdown measure led to a significantly decreased infection growth rate during this period [3]. However, COVID-19 patients were also encountering unforeseen problems, the inability to go offline to seek help. The demand for using social media to access and seek help is unprecedented [2, 4–7]. Weibo, the leading Chinese social media with over 516 million active users each month [8], is an essential platform for seeking help in a crisis [9, 10]. During the lockdown, the Wuhan area was flooded with numerous help-seeking posts about relief materials and medical on Weibo [8].

It is, therefore, worth exploring help-seeking posts on social media in this special situation. On the one hand, the help-seeking posts reflected the offline difficulties and the real emotions of help-seekers during the lockdown. On the other hand, the online behavior of other users on social media in response and engagement to these help-seeking posts, such as comments and retweets, is a type of help and support in emergencies [10]. The higher level of user engagement provides a greater chance of exposure and public attention, even determining the likelihood of survival for online help-seeker [2]. Previous studies have focused on how content features of help-seeking posts impact user engagement at the beginning of the COVID-19 pandemic [2, 4, 5]. The analysis of the main features is classified based on social support theory (i.e., instrumental support and emotional support). However, they did not consider the relationship between more actual demands of help-seeking (e.g., seeking medicine and food) in the COVID-19 context and user engagement. This relationship reflects what is of the most concern and importance to the help provider in such a crisis. Furthermore, there has been a lack of in-depth exploration on content features of help-seeking posts, such as narrative subject (who is speaking) and narrative mode (how it is being said), which may widely occur and potentially affect the attention and engagement of online users. In addition, emotion is an important driver for the diffusion of help-seeking posts [2, 4, 5]. Many studies have investigated the sentiment analysis of help-seeking posts during the pandemic, demonstrating a trend of prevalent and fluctuating emotions [11–13]. Nevertheless, little research has been conducted on the emotional display of posts with different features and requests.

Given that, this study investigated the concrete demands and features of help-seeking posts during the lockdown, including help-seeking typology, narrative mode, narrative subject, and emotional valence. It expands in three ways based on this past work: (1) concentrating on more contextualized content and features of help-seeking posts on the COVID-19 outbreak, which adapts the framework of the social support theory and increases more practical implications; (2) examining what emotional valence are presented by different content features, which builds the bridge between separated previous studies of features and emotion; (3) exploring how features of help-seeking posts influence user engagements on social media (comments and retweets). Our research on help-seeking posts will provide valuable insights into the help-seeking patterns on social media. These insights can be used to develop effective message strategies for help-seekers in the future. Additionally, our findings can inform governments and organizations about the urgent needs of people in the offline isolation period, as well as provide guidance on how to stabilize public sentiment in response to the similar public health emergency in the early stages.

## Methods

### Data collection

The raw data was initially obtained through web crawlers that collected Weibo posts with *help-seeking* hashtags including the *COVID-19 help-seeking* (新冠肺炎求助), *Wuhan help-seeking* (武汉求助), lockdown help-seeking (封城求助), and *pneumonia patients help-seeking* (肺炎患者求助) from IP addresses located in Wuhan. The data range was from January 23, 2020, at 00:00 to March 24, 2020, at 00:00, encompassing the period from the commencement of the Wuhan lockdown until the Wuhan government officially allowed the green health code for travel. To avoid missing any other help-seeking posts, we as well conducted supplementary keywords searching. The keyword of *help-seeking*(求助) was set as the necessary keyword and the *pneumonia* (肺炎), *coronavirus* (冠状病毒), *novel pneumonia* (新型肺炎), *coronavirus pneumonia* (新冠肺炎) and *pandemic* (疫情) which appear at least once were set as the necessary keywords to supplement the sample. In total, 6221 posts were collected. After removed duplicates and invalid posts that did not contain help-seeking information (see Additional file 1 for the exclusion criteria), eventually, a sample of 2,055 valid help-seeking posts was retained for content coding.

### Operationalization of variables

#### Help-seeking typology

Previous research on online help-seeking posts was operationalized based on the framework of social support theory [9, 14, 15]. However, given the period of special epidemics, “Seeking medical help” and “Seeking relief material” was a great concern in public opinion on Weibo [8]. Based on previous studies, help-seeking typology contains medical help-seeking, material help-seeking, mental help-seeking and informational help-seeking.

#### Narrative mode

According to the content of narrative types, the prior study divided message content into narrative and factual [16]. In the real field, people’s posts are likely to take a mixture of informational (factual) and storytelling (narrative) appeals [17], and some posts are briefer and do not constitute any one mode. Thus, narrative mode is composed of the narrative, the factual, mixed and others.

#### Narrative subject

People with difficulties or patients are both the subject of online information seeking and the subject of the narrative [18–20]. In addition, family (representing close kinship) and friends (representing close social relationships) play vital roles and seek information for people who encounter medical issues [21]. Notably, social media messages broadly impact individuals and groups who connect to the online world to seek information from the public [22]. Therefore, narrative subject was operationalized into patients themselves, friends of patients, relatives of patients and people of unrelated patients.

#### Emotional valence

Emotional valence is based on the basic units [12] divided into positive, negative, and neutral.

The measurement scheme was constructed based on previous literature. Detailed definitions of those categories are listed in Additional file 1.

### Inter-coder reliability and data analysis

In this study, narrative mode, narrative subject, and emotional valence were encoded as mutually exclusive nominal variables. As people faced more than one difficulty during the pandemic, help-seeking typology was operationalized as a non-mutually exclusive nominal variable. This manual content analysis was conducted by two trained coders according to the coding scheme. The Cohen’s kappa scores for all codes of content ranged from 0.76-0.94, which reflected good intercoder reliability. Chi-square tests and post hoc tests on the adjusted residuals using Bonferroni correction were conducted to examine the differences in emotional valence expressed across different features of posts. Regarding help-seeking typology as a non-mutually exclusive nominal variable, multiple correspondence analysis was applied in exploring its difference in emotional valence.

Retweets and comments were respectively employed as dependent variables that greatly fit the measurement of user engagement [23] and message diffusion [4]. Given that comments (M = 7.44, SD = 12.19, Skewness = 2, Kurtosis = 3.89) and retweets (M = 7.37, SD = 29.8, Skewness = 14.69, Kurtosis = 331.97), which is heteroscedasticity and non-normal conditional distribution. Thus, we chose negative binomial regression as the most appropriate way for this study [24, 25].

## Results

### Types of help-seeking posts

First, Online help-seeking was categorized into four types. Medical help-seeking (*N* = 2008, 97.7%) was the most frequently help-seeking type, followed by informational help-seeking (*N* = 1183, 57.6%), material help-seeking (*N* = 395, 19.2%) and mental help-seeking (*N* = 12, 0.06%). In term of narrative mode, most posts were adopted by mixed (*N* = 954, 46.4%), followed by narrative (*N* = 757, 36.8%), factual (*N* = 186, 9.1%), others (*N* = 158, 7.7%). Regarding narrative subject, the main number of posts were released by relatives of patients (*N* = 1267, 61.7%), and friend of patients (*N* = 291, 14.2%), patients themselves (*N* = 276, 13.4%), people of unrelated patients (*N* = 221, 10.7%) were relatively low frequency. In addition, the vast majority of post express negative (*N* = 1915, 93.2%) emotion, which far more than neutral (*N* = 72, 3.5%) and positive emotion (*N* = 68, 3.3%).

### Different types of help-seeking posts and emotional valence

As shown in Table 1, the different types of help-seeking significantly affected emotional efficacy (χ^2^(6) = 47.687, *p* < 0.001). More specifically, informational help-seeking posts were more likely to express negative emotions and less likely to express neutral and positive than medical and material help-seeking.

**Table 1:** Number and percentages of emotional valences for help-seeking typology

	Medical	Material	Informational	Mental
Negative	1876 (93.4%) ^a^	373 (94.4%) ^a^	1155 (97.6%) ^b^	10 (83%) ^a^
Positive	68 (3.4%) ^a^	7 (1.8%) ^a^	3 (0.3%) ^b^	0 (0%)
Neutral	64 (3.2%) ^ab^	15 (3.8%) ^ab^	25 (2.1%) ^b^	2 (17%) ^a^

χ^2^(6) = 47.687,* p* < .001 (because the sub-cell of mental help-seeking contains 0, the chi-square test does not include this category); In each row, different letters indicate significant differences between these categories

A χ^2^ test (see Table 2) indicated that different narrative modes had different inclinations of emotional valence (χ^2^(6) = 207.67, *p* < 0.001). Negative emotion was more frequently present in mixed (98.4%) and narrative (92.2%) than factual (86%) and others (74.7%). In turn, factual (14%) and other (13.9%) posts in the expression of neutral emotion were significantly more frequent than mixed (0.7%) and narrative (2.2%).

**Table 2:** Number and percentages of emotional valences for narrative mode

	Narrative	Factual	Mixed	Others
Negative	698 (92.2%) ^a^	160 (86.0%) ^b^	939 (98.4%) ^c^	118 (74.7%) ^d^
Positive	17 (2.2%) ^a^	26 (14.0%) ^b^	7 (0.7%) ^c^	22 (13.9%) ^b^
Neutral	42 (5.5%) ^a^	0 (0%)	8 (0.8%) ^b^	18 (11.4%) ^c^

χ^2^(6) = 207.666, *p* < .001 (because the sub-cell of mental help-seeking contains 0, the chi-square test does not include this category); In each row, different letters indicate significant differences between these categories

In relation to the presence of emotional valence (see Table 3), there was a significant effect on the type of narrative subject (χ^2^(6) = 141.1, *p* < 0.001). Concretely, the help-seeking posts by patients themselves and relatives of patients featured a significantly greater proportion of negative emotion than the posts by the friends of patients and people of unrelated patients.

**Table 3:** Percentages of emotional valences for narrative subject

	Patients themselves	Friend of patients	Relatives of patients	People of unrelated patients
Negative	266 (96.4%) ^a^	261 (89.7%) ^b^	1221 (96.4%) ^a^	167 (75.6%) ^c^
Positive	8 (2.9%) ^a, b^	16 (5.5%) ^b, c^	22 (1.7%) ^a^	26 (11.8%) ^c^
Neutral	2 (0.7%) ^a^	14 (4.8%) ^b^	24 (1.9%) ^a^	28 (12.7%) ^c^

χ^2^(6) = 141.1 *p* < .001. In each row, different letters indicate significant differences between these categories

### Different types of help-seeking posts and social media user engagement

To examine the relationship between help-seeking typology and comments and retweets, we conducted respectively two negative binomial regressions, one dependent variable for comments and the other for retweets. The help-seeking typology, Narrative mode, and Emotional valence were included as independent variables. The result of Table 4 showed that the informational help-seeking (B = 0.52, *p* < 0.001) and the material help-seeking (B = 0.19, *p* < 0.05) significantly increased the likelihood of comment, indicating that the help-seeking post was likely to be commented 1.68 (*e*^0.52^) times more if it posted with informational help-seeking content, and 1.21 (*e*^0.19^) times slightly more if it posted with material help-seeking content. The other two help-seeking types were not significant in comments. Interestingly, four help-seeking types were all significant in the count of retweets. Both informational help-seeking and medical help-seeking types increased 1.77 (*e*^0.57^) and 1.74 (*e*^0.55^) times, respectively, in the likelihood of retweets. Material help-seeking type had a relatively less increase of 1.18 (*e*^0.16^) times. Conversely, the help-seeking post was likely to be retweeted 68% (1-* e*^−1.15^) less if it was posted with mental help-seeking content.

**Table 4:** Identification of predictors of the number of comments and retweets using a negative binomial regression modal

	Comments				Retweets			
	B	Expe (B)	Wald Chi-Square	*p*	B	Expe (B)	Wald Chi-Square	*p*
**Help-seeking typology**								
Medical	-0.18	0.84	1.13	.288	0.57	1.77	9.23	.002
Material	0.19	1.21	9.34	.002	0.16	1.18	6.49	.011
Informational	0.52	1.68	101.04	.000	0.55	1.74	103.07	.000
Mental	0.05	1.05	0.02	.878	-1.15	0.32	11.25	.001
**Narrative mode**								
Narrative	0.43	1.54	15.08	.000	1.50	4.47	160.90	.000
Factual	0.41	1.51	10.10	.001	1.49	4.43	116.57	.000
Mixed	0.63	1.87	31.32	.000	1.88	6.53	259.24	.000
Others	0^a^	1.00			0^a^	1.00		
**Narrative subject**								
Friends of patients	-0.87	0.42	87.10	.000	-0.64	0.52	45.60	.000
Relatives of patients	-0.29	0.75	16.58	.000	-0.18	0.84	5.92	.015
People of unrelated patients	-0.27	0.76	6.36	.012	0.47	1.60	20.07	.000
Patients themselves	0^a^	1.00			0^a^	1.00		
**Emotional valence**								
Negative	-0.73	0.48	27.40	.000	-0.54	0.59	13.16	.000
Positive	-0.52	0.58	8.61	.003	-1.20	0.30	34.88	.000
Neutral	0^a^	1.00			0^a^	1.00		

^a^as referential group

With regard to the narrative mode, mixed type (B = 0.63, *p* < 0.001, *e*^0.63^ = 1.86), narrative type (B = 0.43, *p* < 0.001, *e*^0.43^ = 1.54) and factual type (B = 0.41, *p* < 0.01, *e*^0.41^ = 1.51) were expected to receive more count of comments than other types. Similarly, compared with other type, posts employed mixed type (B = 1.88, *p* < 0.001), narrative type (B = 1.50, *p* < 0.001) and factual type (B = 1.49, *p* < 0.001) increased significantly retweets, each engendering 6.53 (*e*^1.88^),4.47 (*e*^1.50^) and 4.43 (*e*^1.49^) times more than other types. By adjusting the reference type, mixed type in comments and retweets were significantly different from others (all *p* < 0.001). Narrative type was not significantly different from that factual type both in comments (*p* = 0.115) and retweets (*p* = 0.156).

For narrative subject, the expected log comment count of posts by patients themselves was significantly more than posts by friend of patients (B = -0.87, *p* < 0.001, *e*^−0.87^ = 0.42), posts by relatives of patients (B = -0.29, *p* < 0.001, *e*^−0.29^ = 0.75) and posts by people of unrelated patients (B = -0.27, *p* < 0.05, *e*^−0.27^ = 0.76). Similarly, in term of be retweeted, compared with posts by patients themselves, posts by friend of patients received less attention (B = -0.0.64, *p* < . 001, *e*^−0.64^ = 0.52), posts by relatives of patients (B = -0.18, *p* < 0.05, *e*^−0.18^ = 0.84) afterward. However, unlike the former result, posts by unrelated patients received more retweets than by patients themselves (B = 0.47, *p* < 0.001, *e*^0.47^ = 1.60).

To explore how emotional valence affects help-seeking diffusion. The result showed that posts contained neutral emotion is likely to received more comments than positive ones (B = -0.52, *p* < 0.01, *e*^−0.52^ = 0.58) and negative ones (B = -0.73, *p* < 0.001, *e*^−0.73^ = 0.48). Comparably, the expected log retweet count of posts contained neutral emotion was significantly more than that contained negative (B = -0.54, *p* < 0.001, *e*^−0.54^ = 0.59) and positive emotion (B = -1.2, *p* < 0.001, *e*^−1.2^ = 0.3). It is slightly different that positive posts are likely to receive more comments, while negative posts are more likely to be retweeted.

## Discussion

This study contributed to understanding contextualized types of online help-seeking during the first COVID-19 lockdowns, the features of these help-seeking posts, and explore how these types and features influenced user engagement in help support. Overall, our result showed that most help-seeking posts were for medical information and expressed negative emotions. Posts with mixed narrative mode and by relatives had more negative emotions. Posts seeking information, with a mixed narrative style and neutral emotions received more comments, while posts seeking medical information, with a mixed narrative style, neutral emotions, and released by people unrelated to the patient received more retweets.

### What did people demand the most help with during the first COVID-19 lockdown?

This study found that during the first COVID-19 lockdown, the most urgent demand was for patients to seek medical help [7]. Among medical help-seeking posts, in addition to the demand for COVID-19 related medical assistance, people also urgently seek help for the treatment of other diseases, especially those that require long-term treatment or have a strong dependence (e.g., hemodialysis and insulin). Due to limited access to medical resources and in-person healthcare caused by restrictions of the lockdown, people delayed diagnosis and treatment of medical conditions. As a result, people had to turn to seek medical help online. Government and medical institutions were not adequately prepared and supplied medical resources to people stuck in case of emergencies [26].

Additionally, the help-seeking posts also presented a robust demand to seek information. Switching to the help-provider point, the negative binomial regression showed that people are more likely to retweet medical and informational help-seeking posts. One possible reason for higher retweets is closely related to the volume of these types of posts. Given that the media has limited exposure resources, a greater number of posts implies more exposure and may result in other types of seeking help being put back or overlooked. it is undeniable that both the number of posts and user engagement suggest that the public considers both medical and informational issues to be a priority.

### What were the features of help-seeking posts? And what the features win more public online engagement?

In terms of features of help-seeking posts, those posts were mainly published by others, especially by the relatives of patients. Meanwhile, mixed (narrative and factual) and narrative dominate the expression. From the perspective of online medical consultation, patients may lose the ability to post messages for help due to the limitations of their illness. Meanwhile, patients are more likely to seek help through surrogate seekers (family and friends) who describe the problem in third-person narratives [27].

Going further, the result (Table 4) showed that help-seeking posts by patients themselves may receive more comments and a relatively high number of retweets compared to help-seeking posts by others. It is in line with previous studies that a first-person message triggers more retransmission [2] and gets more interaction [28] than a third-person message. Posting by patients themselves and posting by others represent various levels of perceived psychological distance, which cause different levels of attention [2]. Posts by themselves offer closer psychological distance and a stronger effect of “narrative distance” on emotional involvement [29] than that by others. It is to be noted that the posts by people of unrelated patients received most retweets than others, which is seem to contradict the preceding inference. However, we found that the posts by people of unrelated patients were not first-hand information, instead, these people filtered the information that they are concerned about from the numerous help-seeking information. Thus, they play the role of “gatekeeper” or “filters” to exclude the posts that may be not vulnerable to attention. Given that, patients posting their own requests for help is a relatively effective way of gaining public attention.

Consistently to a recent study, posts with the narrative received more comments and retweets than posts that did not adopt any narrative mode [30], especially posts with the mixed mode worked best. More specifically, in the lockdown situation, the emotional involvement and reactions from the narrative type [31, 32] and veracity and information from the factual type [31] are both critical drivers in influencing the public, and the combination of the two produces a stronger cumulative effect.

Our study aligns with previous studies that during this period of the COVID-19 lockdown, help-seeking posts were loaded with high-arousal emotions [4], especially negative emotions being the dominant ones [7, 12, 33, 34]. During this special situation, people who sought help were more likely to have negative emotions (e.g., anger, fear, and sadness) rather than positive emotions (e.g., encouragement and hope) [13]. Regarding retweets and comments, the neutral post has higher levels of user engagement than positive and negative ones, which ran contrary to the recent study [25] but consistent with the study in the COVID-19 context [25]. Emotion and reason are the twin engines of persuasiveness and responsiveness in online communication [35]. Neutral information has a higher degree of credibility than subjective information [36]. People are more prone to adopt a rational perspective in assessing authenticity and solving the problems in help-seeking posts during the early time of the epidemic.

### How do help-seeking posts present emotions?

The different features of help-seeking posts showed various tendencies in terms of emotions. Negative emotions appeared more frequently in the posts that were informational help-seeking, mixed (factual and narrative) expressions, posted by patients themselves and relatives of patients. Practically, informational demand may be psychologically perceived as most urgent for help-seeker. Since COVID-19 was an unknown disease, people were lack of sufficient and accurate information about the outbreak and how to deal with it [37]. This fear of the unknown and uncertainty inspires more negative emotions. In addition, patients themselves and their relatives have stronger negative emotions. The patients as the sufferer of COVID-19 and the relatives as the people who live together and are close to the patient both experience negative feelings such as disease pain, despair, and anger most directly. Given that, we suggest that an effective way to stabilize public sentiment during a public health crisis is to release adequate and effective information promptly and to offer appropriate care to the patient and their families. To explore the relationship between narrative modes and emotion, we found that negative emotion occurred more in mixed and narrative posts than in factual posts. Consistently, narratives are theorized to work based on emotion compared to non-narratives [16, 31, 38, 39]. However, based on the above result about the limited role of emotions on comments and retweets, our research found that emotion maybe not be the leading variable that causes the persuasive effect in narrative communication during the epidemic. Future research could explore whether narrative and emotion interact in driving engagement to help-seeking posts.

### Limitations

The limitation of this study is inescapable. Firstly, while the quantity of comments is one indicator of user engagement in help-seeking posts, the content of the comments is an even more significant reflection of interaction and help. Future research could further explore the concrete information of comments on help-seeking posts through content analysis or qualitative methods. The second is a simplistic categorization of emotions. It would be more valuable to examine the relationship between content features and other detailed emotions, particularly negative ones, such as anger, fear, and frustration, that more frequently occur in crises. Thirdly, conditioned by manual coding, other media content features, such as images, were not included, which may miss factors that potentially affect public attention. Finally, although we used multiple methods to search and collect help-seeking posts, there may still be some posts that were not retrieved or filtered by the platform. Therefore, future research would benefit from obtaining raw data directly from the company of the platform.

## Conclusion

COVID-19 is still an unsolved and dynamic problem nowadays. During the period of the first COVID-19 lockdowns, the unprepared lockdown led people to rely more heavily on social media for help. This study explores the help-seeking typology and content features (narrative mode, narrative subject, and emotional valence) of help-seeking posts during this period. Meanwhile, it investigates how these features are reflected in emotional valence and driven online user engagement. In the practical implication, the results suggest that (1) the medical and the information are primary concerns to both the help-seeker and help-provider; (2) individually, adopting narrative strategies (especially mixed of narrative and factual) and posting help messages by themselves may gain more online comments and retweets; (3) for the governor, timeously posting information and offering assistance to patients and their families is an effective way of stabilizing public sentiment.

Theoretically, we build on the online help-seeking analysis research in the COVID-19 period, developing a more practical and contextualized research framework, and also provide insight into the investigation of the relationship between emotional valence and content features of help-seeking posts. This study provides recommendations for individuals seeking help on social media during comparable public health crises.

## Supplementary Information

**Additional file 1.** Table for manually filtered criteria.

## Data Availability

The data used in the current study are available from the corresponding author on reasonable request.

## Abbreviation

COVID-19: Coronavirus disease 2019
