# Supplementary material for: “Help Us!”: a content analysis of COVID-19 help-seeking posts on Weibo during the first lockdown
Source: BMC Public Health. 2023 Apr 19;23:710. doi: 10.1186/s12889-023-15578-y (PMC10113719; doi:10.1186/s12889-023-15578-y)
Supplement: Supplementary file 1 — Additional file 1. Table for manually filtered criteria. [file 12889_2023_15578_MOESM1_ESM.docx]

**Appendix**

| *Table for* *Manually Filtered Criteria* | | |
| --- | --- | --- |
|  | Criteria | Example |
| The inclusion | The user is asking for assistance or advice with concrete requests. | “#COVID-19 Help-Seeking# #Pneumonia Patients Help-Seeking [Super Topic]# #Wuhan# An elderly COVID-19 patient was discharged on February 20th and placed in isolation at a hotel. On March 4th, the patient tested positive again during a follow-up examination, and on March 7th, the patient experienced symptoms of bloody stools and difficulty breathing. The patient has been registered with the community and the mayor's hotline, but still hasn't been admitted to a hospital. Does anyone know what to do in this situation??” |
| The exclusion  (Regarding uncertain references) | No actual content | “#Pneumonia Patients Help-Seeking[Super Topic]#@People's Daily Online @Voice of Hubei Radio @Popular Application Apps @Wuhan Traffic Radio @CNR News @China Meteorological Administration” |
|  | Irrelevant content | “#Pneumonia Patients Help-Seeking[Super Topic]# Emoji” |
|  | Response to help | “Thank you, everyone. With your help, my father has been admitted to the Tongji Hospital, and his condition is relatively stable now. Thank you very much for your reposts and comments. Please take precautions and let's win this battle together. I hope this topic can be cleared soon.#COVID-19 Help-Seeking#” |
|  | Offer help | “#Pneumonia Patients Help-Seeking[Super Topic]# Below are the green channels and guarantee methods for medical supplies, as well as the contact information for hospitals accepting assistance. Please help spread the word.” |
|  | Only have Comments | “On the 25th day of the lockdown: Hopefully in the future there will be a monument erected in memory of those who have passed away, to commemorate the pain and shame of this city. #Pneumonia Patients Help-Seeking[Super Topic]#” |
|  | No concrete requests | “Asking for help with real identity number #COVID#” |

*Note.* To test the accuracy of exclusions, 100 posts were conducted. The Cohen’s kappa score is 1.00. All examples come from posts in our research corpus (translated from Chinese).

| *Table for Concepts and Corresponding Variables* | | | |
| --- | --- | --- | --- |
| Variable | Category | Definition | Example ^a^ |
| Help-seeking typology ^b^ | Medical | Medical help-seeking means seeking medical supplies, medicine, and advice related to a medical condition or health issue [1]. | “Currently, medication is in urgent shortage, and the situation has worsened rapidly in the past two days, with breathing difficulties and inability to breathe properly. Urgent medication treatment is needed!!” |
|  | Material | Material help-seeking involves asking for daily necessities or everyday items, such as food supplies, and money [2]. | “Life is getting harder, there's not enough food at home, and I dare not go to the supermarket to buy things because many items are out of stock!” |
|  | Mental | Asking for psychological help, such as empathy or other positive affection toward people who suffer from misfortune [3]. | “Although she still had a fever of 38 degrees in the morning, she was physically okay, but psychologically not doing well. She's locked herself in the room alone, and I feel like she's going crazy being cooped up. I need to find something for her to do.” |
|  | Informational | Seeking for information related to updates on the epidemic, government measures, medical resources, safety precautions, living guarantees, and social assistance [4]. | “What is our emergency plan if Wuhan experiences another peak in the epidemic?”  “I would like to ask if all residential communities are managed in this way during the epidemic period, with only one elevator in operation for each building's public facilities? Does having only one elevator increase the risk of infection or does it reduce the risk of infection?” |
| Narrative mode | Narrative | Refers to stories that people tell and use, consisting of anecdotes and personal stories with plots [5, 6]. | “Due to the epidemic and the city lockdown, I am writing this letter to ask for help with tears in my eyes for my son. Hello, everyone with love in your heart! My name is X, I am from Hubei. My son, X, is 4 years old and has leukemia. He has been receiving chemotherapy at Wuhan Union Hospital for over 7 months now. We have used up all our savings and borrowed money from everywhere. The doctors said that we need another 300,000 yuan for the next stage of treatment. This is a huge amount for an ordinary family like ours. Because of the strict epidemic control measures in Wuhan, we can't go back home to raise funds and the expensive medical expenses make it very difficult for us to continue with the treatment. Sometimes, we even thought of giving up the treatment, but a life is at stake. We have no choice but to seek help and plead with kind-hearted people for donations. Even if you can only share this letter once, or donate just one penny, it means the world to my child.” |
|  | Factual | Refers to objectively presenting personal information, personal reasons, symptom descriptions, and diagnosis results [6]. | “Name: X, Age: 63, City: Wuhan, Neighborhood/Community: X , Date of illness onset: January 31^st^, Contact number: X, Other emergency contact: X Description of illness: On January 31st, the patient had low fever for the first time. On February 2nd, she went to Hubei Third People's Hospital fever clinic for blood tests and CT scans, and received injections in the clinic. From February 2nd to February 4th, she had high fever after injections and requested nucleic acid testing. On February 5th, the nucleic acid test result was positive, confirming the new coronavirus infection. The patient currently has high fever, body aches, anorexia, difficulty breathing, and gradually decreasing consciousness. We request that the community contact the hospital to arrange a hospital bed for treatment.” |
|  | Mixed | Includes both the narrative plot and objectively presenting personal information, personal reasons, symptom descriptions, or diagnosis results [7]. | “Name: X, Age: 46, City: Wuhan, Neighborhood/Community: X, Date of illness onset: 1.18, Contact number: X, Other emergency contact: X  Description of illness: Requesting assistance to forward. My father, 46 years old this year, is a bus driver and a regular employee of the Bus Four Company. He had a slight cold since January 18th, but didn't pay much attention to it and still worked for two more days. On January 20th, he started to have a fever and felt weak all over with a floating sensation. He went to Wuhan Fifth Hospital for treatment with injections and medicine, but there was no improvement. He had high fever every night and diarrhea, which persisted until February 2nd. Later, he took a nucleic acid test, and the result was positive. We thought he could be admitted to the hospital, but the hospital kept pushing and said there were no beds available. Should we really wait for him to die at home with no one to help us?” |
|  | Others | Except factual, narrative, and mixed modes. | “Time is life. Can we help them by giving them medicine?” |
| Narrative subject | Friends of patients | Seeking help by mentioning or reporting friends including colleagues, neighbors, and classmates. | “The patient is my friend and a junior high school classmate. He has had a relapse of glioma and has been paralyzed and unconscious at home for several days. His mother, who is over 60 years old, is the only caregiver for him, as well as raising a daughter who is less than 6 years old. He is seeking treatment at a non-COVID-19 hospital and hoping that everyone can help him.” |
|  | Relatives of patients | Seeking help by mentioning or reporting relatives, parents, and partners. | “My father has been experiencing fever for 10 days now, and is currently quarantined in a hotel arranged by the local street office. However, my father had a serious head injury in a car accident in 2007, and developed epilepsy as a result. He is now lying on the floor in the hotel and finding it very difficult to move. I hope that any relevant authorities who see this can help my father get admitted to the hospital as soon as possible!” |
|  | People of unrelated patients | Seeking help by mentioning or reporting people who are not kinship (parents, relatives) or socially connected (friends, colleagues, relatives, neighbors). | “Can everyone who sees this also post to help the residents of Baibuting?” |
|  | Patients themselves | Ask for help in the first person. | “I am a suspected case of viral pneumonia and also a close contact.” |
| Emotional valence | Negative | Conveys negative emotions such as sadness, frustration, and anger. | “Please help my hometown Wuhan! Help these poor people who are diagnosed but have nowhere to seek medical treatment! One person's inability to be admitted to the hospital for isolation and treatment has caused the entire family to be infected, but they can only wait for death at home, how desperate it is!” |
|  | Positive | Conveys feelings of trust confidence, hope, and the eagerness to return kindness [8]. | “Can everyone please scan this QR code when convenient, or simply search for Wuhan Charity Federation? Any amount of money will do, even just a few dollars. I always believe that there are always more kind-hearted people in this world.” |
|  | Neutral | Does not convey any strong emotions or feelings [9]. | “After one day of treatment, her father's condition has become more severe with signs of heart failure. The attending doctor has issued a critical illness notice and currently, the Fifth Hospital is unable to provide further treatment. Please consider the severity of her father's current condition and help arrange for him to be transferred to a hospital with better medical conditions for rescue. Thank you for your attention.” |
| *Note*. ^a^ All of the examples presented are excerpts from the full posts in our research corpus (translated from Chinese) and All personally identifiable information involved in the case examples has been replaced with X for anonymity. ^b^ Help-seeking typology was operationalized as a non-mutually exclusive nominal variable. | | | |

**References**

1. Han X, Wang J, Zhang M, Wang X. Using Social Media to Mine and Analyze Public Opinion Related to COVID-19 in China. Int J Env Res Pub He. 2020;17:2788.

2. Wang J, Zhou Y, Zhang W, Evans R, Zhu C. Concerns Expressed by Chinese Social Media Users During the COVID-19 Pandemic: Content Analysis of Sina Weibo Microblogging Data. J Med Internet Res. 2020;22:e22152.

3. Luo C, Li Y, Chen A, Tang Y. What triggers online help-seeking retransmission during the COVID-19 period? Empirical evidence from Chinese social media. PloS One. 2020;15:e0241465.

4. Karmegam D, Mapillairaju B. What people share about the COVID-19 outbreak on Twitter? An exploratory analysis. BMJ Health Care Info. 2020;27:e100133.

5. Hinyard LJ, Kreuter MW. Using Narrative Communication as a Tool for Health Behavior Change: A Conceptual, Theoretical, and Empirical Overview. Health Educ Behav. 2007;34:777–92.

6. Shen F, Sheer VC, Li R. Impact of Narratives on Persuasion in Health Communication: A Meta-Analysis. J Advertising. 2015;44:105–13.

7. Stubb C. Story versus info: Tracking blog readers’ online viewing time of sponsored blog posts based on content-specific elements. Comput Hum Behav. 2018;82:54–62.

8. Chen A, Ng A, Xi Y, Hu Y. What makes an online help-seeking message go far during the COVID-19 crisis in mainland China? A multilevel regression analysis. Digit Health. 2022;8:20552076221085060.

9. Mittal R, Mittal A, Aggarwal I. Identification of affective valence of Twitter generated sentiments during the COVID-19 outbreak. Soc Netw Anal Min. 2021;11:108.
